# Are immunosenescent T cells really senescent?

**DOI:** 10.1111/acel.14300

**Published:** 2024-08-07

**Authors:** Helena Slaets, Naomi Veeningen, Peter L. J. de Keizer, Niels Hellings, Sven Hendrix

**Affiliations:** ^1^ Neuro‐Immune Connections and Repair Lab, Department of Immunology and Infection Biomedical Research Institute, Hasselt University Diepenbeek Belgium; ^2^ UMSC–University MS Center, Campus Diepenbeek Diepenbeek Belgium; ^3^ Center for Molecular Medicine University Medical Center Utrecht Utrecht The Netherlands; ^4^ Institute of Translational Medicine, Medical School Hamburg Hamburg Germany

**Keywords:** exhaustion‐T‐lymphocytes, immunosenescence‐aging, senescence, T‐cells

## Abstract

Loss of proper T‐cell functioning is a feature of aging that increases the risk of developing chronic diseases. In aged individuals, highly differentiated T cells arise with a reduced expression of CD28 and CD27 and an increased expression of KLRG‐1 or CD57. These cells are often referred to as immunosenescent T cells but may still be highly active and contribute to autoimmunity. Another population of T cells known as exhausted T cells arises after chronic antigen stimulation and loses its effector functions, leading to a failure to combat malignancies and viral infections. A process called cellular senescence also increases during aging, and targeting this process has proven to be fruitful against a range of age‐related pathologies in animal models. Cellular senescence occurs in cells that are irreparably damaged, limiting their proliferation and typically leading to chronic secretion of pro‐inflammatory factors. To develop therapies against pathologies caused by defective T‐cell function, it is important to understand the differences and similarities between immunosenescence and cellular senescence. Here, we review the hallmarks of cellular senescence versus senescent and exhausted T cells and provide considerations for the development of specific therapies against age‐related diseases.

## Graphical abstract

This review delineates the molecular hallmarks of cellular senescence and examines whether they are evident in aging‐associated T cells referred to as immunosenescent T cells and exhausted T cells.

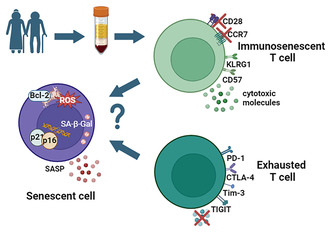

**Abbreviations**
APC: antigen‐presenting cells
CAR T cells: chimeric antigen receptor T cells
CCR7: C‐C chemokine receptor type 7
CM T cells: Central Memory T cells
CMV: cytomegalovirus
CTLA‐4: cytotoxic T lymphocyte‐associated protein 4
DDR: DNA Damage Response
DNA‐SCAR: DNA segments with chromatin alterations reinforcing senescence
EM T cells: Effector Memory T cells
HGF: hepatocyte growth factor
HIV: human immunodeficiency virus
IL‐1: interleukin‐1
KIR: killer immunoglobulin‐like receptors
KLRG‐1: killer‐cell lectin‐like receptor G1
LMNB1: lamin‐B1
MiDAS: mitochondrial dysfunction‐associated senescence
NRF1: nuclear respiratory factor 1
PD‐1: programmed cell death protein‐1
PHA: phytohemagglutinin
PMA: phorbol 12‐myristate‐13‐acetate
ROS: reactive oxygen species
SA‐b‐Gal: senescence‐associated beta‐galactosidase'
SAHF: senescence‐associated heterochromatin foci
SASP: senescence‐associated secretory phenotype
TAF: Telomere‐associated DNA damage foci
TE T cells: Terminal Effector T cells
TEMRA cells: Terminal Effector T cells that re‐express CD45RA
TFAM: mitochondrial transcription factor A
TIGIT: T‐cell immunoglobulin and immunoreceptor tyrosine‐based inhibitory motif (ITIM) domain
TIM‐3: T cell immunoglobulin mucin‐3

## 1 INTRODUCTION

The incidence of age‐related diseases such as Alzheimer's Disease, diabetes, cardiovascular disease, and cancer rapidly rises. Impaired or maladapted immune responses are common in age‐related diseases (Isobe et al., 2017). In addition, older individuals are more vulnerable to novel infections (Zheng et al., 2020). As the global population is aging, this poses a significant burden on healthcare systems worldwide. Therefore, several recent studies have investigated the aged immune system and tested different strategies to protect the elderly from age‐related diseases and infections.

The cells in our body are constantly exposed to various stressors, including reactive oxygen species, toxins, DNA damage, and strong mitogenic signals induced by oncogene expression. When the damage to a cell cannot be repaired, it may undergo apoptosis or enter a state known as *cellular senescence* (Gorgoulis et al., 2019). This stable state of cell cycle arrest assures that the dysfunctional cell ceases proliferation. Senescence is an important mechanism in our body to prevent tumor development. In addition, temporary senescence plays a beneficial role in tissue remodeling during embryonic development, wound healing, the involution of the mammary glands after the cessation of breastfeeding, and the placenta after labor (Demaria et al., 2014; Sirinian et al., 2022).

Once a cell has entered a state of senescence, it may be cleared by the immune system (Xue et al., 2007). However, as the individual ages, the ability of the immune system to effectively eliminate senescent cells begins to decline. As we age, senescent cells accumulate and become a chronic feature within the body. Immune aging or *immunosenescence* affects both the innate and adaptive immune system. This process results in decreased responsiveness to vaccines and increased susceptibility to infections and cancer among the elderly (Fulop et al., 2017).

The aging process has a significant impact on the T cell compartment. The process of thymic involution, along with lifelong exposure to latent viruses, leads to a decrease in overall T‐cell immunity and the emergence of T cells with reduced expression of CD28 and CD27, and increased expression of KLRG‐1 or CD57, which are often referred to as senescent or immunosenescent T cells (Rodriguez et al., 2020). However, whether these T cells are truly senescent cells remains to be determined. Unfortunately, the term “senescent” is ambiguous when applied to T cells.

To make things more complicated, there is another subpopulation of T cells that loses its effector functions after repeated stimulation and becomes exhausted, thereby contributing to decreased T cell‐mediated immunity with aging. Both exhausted T cells as well as immunosenescent T cells are identified with flow cytometry markers and are often referred to as senescent T cells throughout literature. Do these T cells possess the typical hallmarks of senescent cells? This is discussed in the following paragraphs.

## 2 HALLMARKS OF CELLULAR SENESCENCE

The phenomenon of cellular senescence was first discovered by Hayflick & Moorhead (1961), who observed that human fibroblasts in culture could divide around 50 times before they lose the ability to proliferate any further. Since this initial discovery of the ‘Hayflick limit,’ senescence has been described for many somatic cell types. The hallmarks of senescent cells are discussed below and are summarized in panel 1 of Table 2.

### 2.1 Persistent DNA damage and cell cycle arrest

Hayflick's observations have revealed that replicative senescence can be attributed to *telomere attrition* the gradual shortening of telomeres with each cell cycle. Once the telomeres have shortened to a critical point, the remaining DNA is perceived as a double‐strand break, which elicits a DNA Damage Response (DDR). This leads to *Telomere‐associated DDR foci* (*TAFs*), which can be detected as *persistent γH2Ax/53BP1 damage foci* within telomeric DNA (Hewitt et al., 2012). H2Ax is a histone protein that becomes phosphorylated on serine 139 after DNA‐damage, four positions from its C‐terminus, hence leading to the nomenclature *γH2Ax*. Severe DNA damage, such as double‐strand breaks, elicits a DDR aimed at quick and effective DNA repair. However, chronic and unresolved damage can cause γH2Ax to accumulate and persist in stress‐induced cellular senescence. During the repair process after severe DNA damage, proliferation is put on hold. This cell cycle arrest is mediated by the activation of cyclin‐dependent kinase inhibitors such as *p16* ^INK4a^ and *p21* ^CIP1^ (hereafter p16 and p21). The initial stress response is mediated by p53, a transcription factor upstream of p21. p53/p21 signaling initiates senescence, after which the cell reduces p21 and switches to p16 expression, which maintains senescence. While p53 activation induces p21 expression and may, therefore, induce senescence, p21 is also able to induce senescence independent of p53. Therefore, upregulated expression of p16, p21, and also p53 are often used as markers to detect senescent cells (Alcorta et al., 1996). It is important to note that these markers are not exclusively expressed by senescent cells. For example, p53/p21 expression may indicate the intitation of senescence, but could also point to a cell dealing with acute DNA damage (el‐Deiry et al., 1993). In addition, stem cells also upregulate cyclin‐dependent kinases such as p21 to retain a state of quiescence (Terzi et al., 2016). Therefore, additional hallmarks of senescence are needed to detect and study senescent cells.

Besides telomere attrition and *telomere‐associated damage foci* (*TAF*), many other stimuli can induce senescence, such as chemotherapy or radiation‐induced damage, oncogene overexpression, and several types of persistent DNA damage such as double‐stranded DNA breaks, replication fork stalling and failed mismatch repair (Anderson et al., 2019; Gorgoulis et al., 2019). Interestingly, the DNA of senescent cells shows characteristic alterations such as a large foci of DNA damage called ‘DNA segments with chromatin alterations reinforcing senescence’ (*DNA‐SCARS*) (Rodier et al., 2011) and foci of condensed DNA in proliferation‐promoting genes called ‘senescence‐associated heterochromatin foci’ (*SAHF*) (Narita et al., 2003).

### 2.2 Resistance to apoptosis

Following the DDR, a cell has three possible fates: if the damage is successfully repaired, the cell can re‐enter the cell cycle. However, when the damage cannot be repaired (such as after critical telomere attrition), the cell can either undergo apoptosis or remain in permanent cell cycle arrest and become senescent. Some senescent cells upregulate *anti‐apoptotic molecules* such as *Bcl‐2, Bcl‐xL or Bcl‐W*, which could make them more resistant to apoptosis (Yosef et al., 2016). Upregulated expression of these anti‐apoptotic molecules is often used as another hallmark to detect senescent cells. In addition, Bcl‐2 family members can be therapeutically targeted by senolytics such as Navitoclax to eliminate senescent cells. However, other studies found a downregulation of Bcl‐2 in senescent fibroblasts, indicating Bcl‐2 family gene expression in senescence may be cell type and treatment specific. Another mechanism by which senescent cells may become resistant to apoptosis is through the upregulation of *FOXO4*, a transcription factor that binds p53 and mediates pro‐survival effects by upregulating p21 (Baar et al., 2017). Breaking the interaction between FOXO4 and p53 induces apoptosis in senescent cells. The underlying mechanism involves the migration of p53 to the mitochondria where it elicits BAX/BAK‐mediated apoptosis and reduces p21 expression (Baar et al., 2017). In conclusion, several mechanisms may promote resistance to apoptosis in senescent cells, and these mechanisms can be exploited to eliminate them.

### 2.3 Chronic senescence is deleterious for tissue homeostasis

While acute senescence plays a protective role during wound healing and tissue remodeling, things change when senescent cells accumulate and become a chronic feature. Senescent cells accumulate with aging, in sites of pathology, and after chemotherapy or irradiation. Senescent cells such as senescent fibroblasts, epithelial cells and adipocytes secrete a mixture of proinflammatory mediators called the ‘*senescence‐associated secretory phenotype*’ (*SASP*). The SASP consists of inflammatory cytokines, chemokines, growth factors, prostaglandins, matrix metalloproteinases, and exosomal cargo (Basisty et al., 2020). It is important to note that the SASP varies for different cell types, for different inducers of senescence, and even changes over time. However, it typically consists of a subset of proteins elevated in all SASPs, such as the inflammatory cytokines interleukin‐1 alpha (IL‐1α), IL‐6, and IL‐8. Acute release of SASP factors such as CCL2 and CCL7 may attract immune cells toward the senescent cell so that it can be eliminated by macrophages, NK cells or cytotoxic T cells (Kale et al., 2020; Marin et al., 2023). In contrast, chronic exposure to SASP molecules can be harmful to the surrounding cells, which may, in turn, also become senescent, a phenomenon referred to as paracrine senescence (Gonzalez‐Meljem et al., 2018).

As senescent cells accumulate during aging, their SASP may also contribute to the chronic low‐grade inflammation, known as “inflammaging” (Franceschi et al., 2000), that is commonly found in the elderly population. This way, senescent cells may accelerate aging processes throughout the rest of the body. Indeed, selective deletion of senescent (p16‐positive) cells extends the lifespan of mice and attenuates age‐related functional decline of several vital organs such as the liver and the heart (Baker et al., 2016). This study shows that the accumulation of senescent cells during aging negatively affects lifespan and healthspan. In addition, recent independent studies showed that injection of senescent adipocyte progenitor cells, as well as blood exchange from aged into young mice, is sufficient to induce cellular senescence in multiple tissues (Jeon et al., 2022; Xu et al., 2018; Yousefzadeh et al., 2020). Together, these studies indicate that the accumulation of senescent cells during aging reduces lifespan and actively contributes to age‐related functional decline throughout the body.

### 2.4 Changes in cellular organelles

**FIGURE 1:**
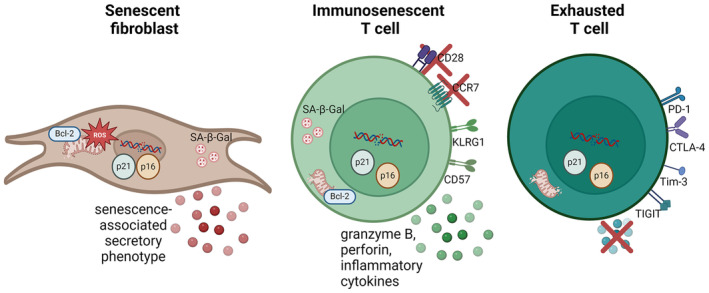
Hallmarks reported for senescent fibroblasts, immunosenescent T cells, and exhausted T cells. *Senescent fibroblasts* have flattened and enlarged cell bodies in vitro. They show DNA damage foci. The cells may upregulate p16 and p21 leading to cell cycle arrest. Senescent fibroblasts may also upregulate Bcl‐family proteins, making them more resistant to apoptosis. They secrete a cocktail of inflammatory molecules termed the SASP. Other hallmarks include dysfunctional mitochondria and high senescence‐associated β‐galactosidase (SA‐β‐Gal) activity. *Immunosenescent T cells* can be identified by a loss of CCR7 combined with re‐expression of CD45RA, in addition to a loss of CD28 and/or CD27. The cells also upregulate natural killer cell‐like receptors such as killer‐cell lectin‐like receptor G1 (KLRG1) and CD57 and acquire cytotoxic functions, producing granzyme B and perforin. Immunosenescent T cells may also upregulate p16 and p21, but it is not clear whether they go into permanent cell cycle arrest. The cells can upregulate Bcl‐2 and become more resistant to apoptosis. They accumulate dysfunctional mitochondria and the majority of them shows high SA‐β‐Gal activity. *Exhausted T cells* lose the ability to perform effector functions such as cytokine production and secretion of cytotoxic molecules. They can be detected by exhaustion markers such as T cell immunoglobulin mucin‐3 (TIM‐3), cytotoxic T lymphocyte‐associated protein 4 (CTLA‐4), and T‐cell immunoglobulin and immunoreceptor tyrosine‐based inhibitory motif (ITIM) domain (TIGIT). Although they may accumulate DNA damage and upregulate expression of p16, only a minority shows high SA‐β‐Gal activity.

Although senescent cells remain metabolically active, they often display an altered morphology and function (Table 1, reviewed in (Huang et al., 2022)). Lamin B1 (LMNB1) is reduced in the nuclear lamina of senescent cells (Freund et al., 2012). The nucleus of senescent cells also displays an altered morphology (Heckenbach et al., 2022). Another typical hallmark of senescence is a reduced functionality of the mitochondria (Korolchuk et al., 2017). Their ability to produce ATP is compromised, and “mitophagy”, the recycling process of damaged mitochondria, is impaired, leading to an accumulation of dysfunctional mitochondria that produce a lot of *reactive oxygen species* (ROS). ROS, in turn, causes damage to cellular structures, including the mitochondria, leading to a positive feedback loop in which cellular damage accumulates. During senescence, a subset of mitochondria may display outer membrane permeabilization, reminiscent of a failed apoptosis response (Victorelli et al., 2023). Release of DNA from the damaged mitochondria or the nucleus (cytoplasmic chromatin fragments), activating TLR or cGAS/Sting signaling also contributes to senescence (Victorelli et al., 2023). The cGAS‐STING pathway leads to SASP production. Interestingly, cells that undergo mitochondrial dysfunction‐associated senescence (MiDAS) display a distinct SASP from cells that undergo genotoxic‐induced senescence. The MiDAS SASP includes IL‐10, TNF‐α and CCL27 but lacks the IL‐1 dependent inflammatory factors (Wiley et al., 2016). Besides mitochondrial dysfunction, senescent cells also show increased numbers of lysosomes, but this does not necessarily reflect an increased degradation process. Instead, the cellular recycling process of autophagy is reduced in senescent cells (Tai et al., 2017). The alterations in lysosomes in senescent cells can be measured as an increased activity of ‘*senescence‐associated beta‐galactosidase*’ (*SA‐β‐Gal*). β‐Gal is an essential enzyme in non‐senescent cells as well, catalyzing the hydrolysis of β‐galactose residues. Through the breakdown of lactose to galactose and glucose, the enzyme is a key energy provider. This lysosomal enzyme accumulates in senescent cells, and its activity can be specifically detected in senescent cells at pH 6.0 (Dimri et al., 1995). It is important to note that none of these hallmarks are specific for senescent cells. Therefore, a combination of several of the hallmarks described above is often used to detect senescent cells (Gorgoulis et al., 2019) (Figure 1 and Table 1, left panel).

**TABLE 1:** Hallmarks of senescence reported for senescent fibroblasts, immunosenescent T cells, and exhausted T cells.

Hallmarks of senescence	Senescent fibroblasts	Immunosenescent T cells^a^ CD28^−^, TEMRA CD57^+^, KLRG‐1^+^	Exhausted T cells PD‐1^+^, CTLA4^+^, TIM‐3^+^, TIGIT^+^
Cell cycle arrest	Yes, often indicated by the upregulation of cyclin‐dependent kinase inhibitors p16 or p21 (Alcorta et al., 1996). No longer proliferate	Conflicting results. Reduced proliferation, upregulation of p16 (protein) and p21 (mRNA) (Henson et al., 2014; Scheuring et al., 2002). Other studies suggest they retain the capacity to proliferate in vivo or after PHA or IL‐15 stimulation ex vivo (Brzezinska et al., 2004; Chiu et al., 2006; Vallejo et al., 2000)	Conflicting results. Yes, indicated by the upregulation of cell cycle inhibitors p16 or p21. No longer proliferate (Janelle et al., 2021). But after repeated immunizations, PD1+ cells were still able to proliferate (Soerens et al., 2023)
DNA damage	Common, although exceptions exist. Evidenced by critically short telomeres, persistent γH2AX DNA damage foci/DNA‐SCARS/TAFs (Allsopp & Harley, 1995; Anderson et al., 2019; d'Adda di Fagagna et al., 2003; Rodier et al., 2011)	Unclear. Evidence of γH2AX upregulation and shortened telomeres, but not clear whether damage/shortening is sufficient to induce senescence (Di Mitri et al., 2011; Effros et al., 1996; Lanna et al., 2014; Monteiro et al., 1996; Riddell et al., 2015)	Yes, evidence of increased γH2AX but no evidence of shortened telomeres (Janelle et al., 2021)
More resistant to apoptosis	Yes, but mechanism may differ between cells. Upregulation of anti‐apoptotic molecules such as Bcl‐2 family members in some senescent cells (Yosef et al., 2016)	Yes. Also upregulation of Bcl‐2 but unclear whether senescent T cells depend on Bcl‐2 for survival (Dumitriu, 2015; Schirmer et al., 1998; Spaulding et al., 1999; Vallejo et al., 2000)	No, upregulation of programmed cell death 1 (PD‐1) makes them more susceptible to apoptosis (Patsoukis et al., 2020)
Senescence‐associated secretory phenotype (SASP)	Yes, secretion of inflammatory cytokines, chemokines, growth factors, prostaglandins, matrix metalloproteinases (Basisty et al., 2020)	Yes, secretion of inflammatory cytokines, but also of cytotoxic molecules granzyme B and perforin (Broux et al., 2012; Echeverria et al., 2015) and increased MMP9 and growth factor production (Rioseras et al., 2021). Depending on the T cell lineage (CD8, Th1, Th2, Th17 cells, Tregs) and the microenvironment, other factors are secreted (Pieper et al., 2014)	No, unable to secrete proinflammatory cytokines upon stimulation (Saeidi et al., 2018)
Altered cellular and nuclear morphology	Yes, in vitro evidence of flattened and enlarged cell bodies, vacuolization, and granularity in the cytoplasm (Huang et al., 2022). In addition, senescence is marked by a loss of lamin B1 (LMNB1) in the nuclear lamina (Baar et al., 2017; Freund et al., 2012) and altered nuclear morphology (Heckenbach et al., 2022)	NI	After repeated in vitro stimulations, exhausted T cells lose expression of LMNB1 mRNA (Janelle et al., 2021)
Loss of normal cell function	Partially (Gerasymchuk et al., 2022)	Partially, defective IL‐2 production, but potent cytotoxic effector functions (Broux et al., 2012; Pereira & Akbar, 2016)	Yes (Saeidi et al., 2018)
Accumulation of dysfunctional mitochondria	Yes, decreased membrane potential, more ROS production (Korolchuk et al., 2017). A subset of mitochondria may display outer membrane permeabilization enabling release of DNA activating cGAS/Sting signaling and SASP (Victorelli et al., 2023). Mitochondrial dysfunction‐associated senescence (MiDAS) SASP lacks IL‐1 dependent inflammatory factors (Wiley et al., 2016)	Yes, for CD8^+^ immunosenescent T cells. No for CD4^+^ immunosenescent T cells (Callender et al., 2020; Henson et al., 2014).	Yes (Scharping et al., 2021).
Beta‐galactosidase activity at pH 6.0	Yes (Dimri et al., 1995)	Yes, majority shows high β‐gal activity (Martinez‐Zamudio et al., 2021)	After repeated in vitro stimulations, around 30% of exhausted T cells show high β‐gal activity (Janelle et al., 2021), however in exhausted T cells directly isolated from elderly individuals, only a minor fraction shows high β‐gal activity (Martinez‐Zamudio et al., 2021)

^a^ Immunosenescent T cells: for simplicity, we included studies on CD28null T cells and TEMRA cells that are CD57^+^ or KLRG1^+^.

Abbreviations: CTLA4, cytotoxic T lymphocyte‐associated protein 4; KLRG‐1, killer‐cell lectin‐like receptor G1; NI, not investigated to our knowledge; PD‐1, programmed cell death protein‐1; ROS, Reactive oxygen species; TEMRA, Terminal Effector T cells that have lost expression of CCR7 and re‐express CD45RA; TIGIT, T‐cell immunoglobulin and immunoreceptor tyrosine‐based inhibitory motif (ITIM) domain; TIM‐3, T cell immunoglobulin mucin‐3; β‐gal, β‐galactosidase.

## 3 CELLULAR SENESCENCE IN T CELLS

Now that we have discussed the typical hallmarks of cellular senescence, it is time to take a closer look at the T cells. T cells show a high potency to expand in response to viruses, bacteria, and other invaders. Those T cells that are specific for latent viruses, common antigens such as pollen, or autoantigens, are likely to proliferate a lot throughout an individual's lifetime. As discussed above, this may lead to telomere attrition and subsequent genotoxic stress. In addition, as T cells migrate towards sites of inflammation, they are likely to be subjected to high amounts of ROS, generated by innate immune cells such as neutrophils and macrophages. This may also cause DNA damage and subsequent senescence. After repeated antigen exposure, two subpopulations of T cells arise that can be distinguished using specific flow cytometry markers. These subpopulations are known as immunosenescent T cells and exhausted T cells. In the following paragraphs, we discuss the changes within the T cell compartment during aging and describe in detail whether immunosenescent and exhausted T cells possess the hallmarks of cellular senescence described above.

### 3.1 The effects of aging on T lymphocytes

The immune system becomes less efficient in protecting us against novel viruses and malignancies during aging. A critical factor leading to decreased immunity is the effect of aging on the T lymphocytes. With increasing age, the hematopoietic stem cells in the bone marrow preferentially give rise to myeloid cells at the expense of the lymphocyte compartment. In addition, the thymus shrinks, and its specialized tissue is replaced by adipose tissue, a process called thymic involution (Xu et al., 2022). As the final steps of T cell development and maturation take place in this organ, thymic involution, together with the reduced lymphopoiesis in the bone marrow, leads to a decrease in the generation of naïve T cells.

#### 3.1.1 T cell differentiation stages

When naïve T cells are released from the thymus, they start to patrol our body. If a T cell recognizes an antigen, the T cell is activated. It starts to proliferate, giving rise to effector cells participating in the immune response against the invading pathogen or the aberrant cell. After clearance of the potential threat, most effector cells die through apoptosis, while others differentiate into memory cells that remain in our body for extended periods. The different T cell subsets can be detected with flow cytometry using specific markers (for commonly used human T cell differentiation markers, see Table 2).

**TABLE 2:** Subsets of naïve and memory human T lymphocytes with commonly used cell surface expression markers.

	CD45RA	CD27	CD28	CCR7
Naïve T cells (N)	+	+	+	+
Central memory T cells (CM)	−	+	+	+
Effector memory T cells (EM)	−	−	+/−	−
Terminal effector T cells (TEMRA)	+	−	−	−

*Central memory cells* (*CM*) are long‐lived cells that preferentially reside in lymphoid tissues and provide protection against recurrent infections (Martin & Badovinac, 2018). CM T cells have self‐renewal capacity in the absence of an antigen. However, as T cells proliferate multiple times throughout an individual's lifetime, the cells gradually lose this self‐renewal capacity.

*Effector memory cells* (*EM*) display reduced proliferation capacity but retain their cytolytic and proinflammatory functions (Martin & Badovinac, 2018). EM T cells reside in peripheral tissues where they can immediately respond to possible threats. They are characterized by a loss of the expression of CD27 and/or CD28. These co‐stimulatory molecules are critical for T cell activation. They interact with CD70 and CD80/86 on antigen‐presenting cells and generate the signal that provides the necessary co‐stimulation to activate T cells. These cells are often referred to as CD28null or CD27 null cells. Loss of CD28 and CD27 is described in ample human studies but has not been extensively studied in mice. EM T cells are further characterized by the loss of C‐C chemokine receptor type 7 (CCR7) expression. CCR7 is also necessary for T cell activation and is involved in T cell homing to secondary lymphoid tissues.

*Terminal Effector T cells* (*TE*) have a late‐stage differentiated phenotype. On top of the loss of both CCR7, CD27, and CD28, they may re‐express CD45RA. When they re‐express CD45RA, they are called TEMRA cells. TEMRA cells are considered to be terminally differentiated or senescent T cells (Henson et al., 2015). The term senescent may be misleading here. Whether these cells fit all the criteria of cellular senescence and whether they are the only population of T cells that can be called senescent is discussed in the following paragraphs.

#### 3.1.2 Aging and T cells

A meta‐analysis found *significant reductions in the frequency of naïve T cells* with aging, most pronounced within the CD8 compartment but also significant within the CD4 population (Rodriguez et al., 2020). In addition, several groups have reported *increased frequencies of CD28 null T cells* in the elderly. Loss of CD28 is believed to be caused by proliferative stress. In vitro, T cells lose expression of their co‐stimulatory molecule upon repeated antigen stimulation (Effros & Pawelec, 1997). In vivo, latent viruses such as the cytomegalovirus (CMV), human immunodeficiency virus (HIV), or autoantigens in autoimmune disease may cause continuous chronic antigen stimulation. Indeed, CD28null cells have been isolated from the peripheral blood of elderly individuals, HIV patients, and autoimmune patients (Broadley et al., 2017; Broux et al., 2012; Echeverria et al., 2015). T cells can be expected to undergo frequent antigen stimulation in all these individuals and, as such, repeated proliferation. In addition, studies investigating CMV‐specific T cells found that most have indeed lost the expression of CD28 (Bano et al., 2019; Vescovini et al., 2004).

### 3.2 The hallmarks of senescence in T lymphocytes

Similar to the ‘Hayflick limit’ described above for senescent fibroblasts, T cells in culture lose their proliferation capacity after a certain number (between 25 and 40) of population doublings (Effros & Pawelec, 1997). This suggests that T cells do indeed have the ability to undergo replicative cellular senescence. In these senescent T cell cultures, most T cells have lost expression of CD28. Therefore, CD28null T cells are generally referred to as immunosenescent T cells.

#### 3.2.1 Cell cycle arrest in immunosenescent T cells

The question remains whether the Hayflick limit described for cultured T cells is also reached by T cells in vivo. A recent study showed that T cells are not intrinsically constrained by cell division limits (Soerens et al., 2023). In this study, mice were immunized, after which the expanded T cell population was transferred to new mice, which were immunized again, and so on. The authors showed that T cells still responded to with proliferation, even after 10 years and 51 successive immunizations. This suggests that T cells do not reach the Hayflick limit in vivo. There are conflicting reports regarding the proliferation capacity of CD28null T cells in vivo. Several studies found that CD28null cells can still proliferate, in vivo as well as after ex vivo stimulation with either PHA, IL‐15, or anti‐CD3 and IL‐2, which indicates they are not in a stable state of cell cycle arrest (Brzezinska et al., 2004; Chiu et al., 2006; Chong et al., 2008; Markovic‐Plese et al., 2001; Vallejo et al., 2000). In contrast, reduced proliferation is detected in human CD28null cells isolated from the peripheral blood compared to their CD28 positive counterparts after CD3 stimulation, but also in response to mitogens that bypass T cell receptor signaling, such as PMA/ionomycin (Henson et al., 2014; Scheuring et al., 2002). The reduced proliferation capacity may partly be due to their decreased ability to synthesize IL‐2 (Thompson et al., 1989).

In addition, the CD28null cells show upregulation of *p16* and *p21*, indicating that these T cells may indeed be in a state of cellular senescence (Scheuring et al., 2002). While p21 is known as a cell cycle regulator, it appears to play a unique role in T cells, namely as a suppressor of autoimmune responses. Overexpression of p21 in T cells reduces the proliferation of autoreactive T cells in a mouse model of lupus without affecting normal T cell responses (Daszkiewicz et al., 2015). Vice versa, deletion of p21 results in autoimmunity, suggesting that p21 plays a role in suppressing T cell‐mediated autoimmunity. Another study also suggests a role for senescence in controlling unwanted T cell responses: human Tregs in culture suppress activated T cells by inducing senescence as indicated by a strong upregulation of p16, p21, and β‐Gal activity. The T cells cease proliferation and downregulate CD27 and CD28 (Ye et al., 2012). These studies suggest that senescence may be essential in controlling unwanted T cell responses.

Nevertheless, as most studies indicate, CD28null T cells may still be able to proliferate in vivo (Brzezinska et al., 2004; Chiu et al., 2006; Chong et al., 2008; Markovic‐Plese et al., 2001; Vallejo et al., 2000). In addition, they become resistant to the suppressive actions of Tregs (Bano et al., 2019; Hoeks et al., 2021). This implies that CD28null T cells, often referred to as immunosenescent T cells, are not all in a state of replicative senescence in vivo.

#### 3.2.2 Telomere attrition in immunosenescent T cells

It is important to note that T cells differ from other somatic cells with regard to the expression of the enzyme telomerase. Telomerase counteracts telomere shortening by the de novo synthesis of telomeric repeats. Most somatic cells, such as fibroblasts, do not express telomerase after birth. Consequently, their telomeres become shorter with every cell division. T cells are an exception. When a T cell recognizes its antigen, T cell receptor activation leads to a transient upregulation of telomerase (Hodes et al., 2002; Maini et al., 1999). This unique property enables clonal expansion of T cells to combat infections and allows long‐lived immunity (Akbar & Vukmanovic‐Stejic, 2007). However, signals through CD28 are required for telomerase induction, so when T cells lose expression of CD28, this results in lower telomerase activity. In addition, telomerase deficiency can lead to reduced expression of CD28, suggesting a feedback loop between both molecules (Matthe et al., 2022). This may lead to CD28null T cells being more susceptible to telomere shortening than CD28^+^ cells.

In addition, another mechanism was recently revealed by which T cells can elongate their telomeres, even when they do not express telomerase themselves (Lanna et al., 2022). T cells can acquire telomeres from vesicles released by antigen‐presenting cells (APC) when they form an immunological synapse (Lanna et al., 2022). Importantly, T cells cultured in the absence of APCs, such as those used to demonstrate the “Hayflick's limit”, may therefore not represent telomere shortening as it occurs upon clonal expansion in vivo. When we look at CD28null cells isolated from human blood, they have shorter telomeres than CD28^+^ T cells from the same individual. In addition, *yH2Ax* is upregulated in TEMRA cells and CD28null T cells (Di Mitri et al., 2011; Henson et al., 2015; Lanna et al., 2014). As these studies used flow cytometry to detect *yH2Ax*, it remains unknown whether immunosenescent T cells display real DNA‐SCARS and TAFs, or merely upregulate small, acute DNA damage. In addition, while CD28null cells have *shorter telomeres* than CD28^+^ cells from the same donor, they are still intermediate in length (Di Mitri et al., 2011; Effros et al., 1996; Monteiro et al., 1996; Riddell et al., 2015). These findings suggest that the telomeres of CD28null T cells do not reach a length that is so critically short that it induces replicative senescence.

#### 3.2.3 Immunosenescent T cells and resistance to apoptosis

Similar to the observations in some senescent fibroblasts that become highly resistant to apoptosis, upregulation of the anti‐apoptotic molecule *Bcl‐2* is also reported in CD4^+^CD28null T cells (Schirmer et al., 1998). However, the latter observation was done on T cell clones, and it remains to be determined whether CD28null cells in vivo also show upregulation of Bcl‐2 or its family members. Several studies showed that both CD4^+^ and CD8^+^ CD28null T cells are more resistant to apoptosis in response to in vitro stimuli such as Fas stimulation, heat, or IL‐2 withdrawal (Dumitriu, 2015; Schirmer et al., 1998; Spaulding et al., 1999; Vallejo et al., 2000) compared to CD28^+^ cells. If CD28null cells are also highly resistant to apoptosis in vivo, they may persist for a person's lifetime, highlighting the importance of understanding the functional changes in immunosenescent T cells.

#### 3.2.4 Gain of functions in immunosenescent T cells

A typical hallmark of senescent cells is the release of the SASP: Senescent cells continuously secrete a multitude of inflammatory mediators. The content of the SASP changes over time and depends on the context and cell type. Similarly, CD28null T cells secrete high levels of inflammatory cytokines. In addition, with the loss of CD28 expression, T cells appear to acquire additional inflammatory functions. CD4^+^CD28null cells start to produce the *cytotoxic molecules granzyme B and perforin*, which are normally only expressed by CD8^+^ T cells and NK cells (Broux et al., 2012; Echeverria et al., 2015). Although T cell receptor signaling is diminished in CD4^+^ T cells from aged individuals, the cells preferentially differentiate into effector cells instead of memory cells. A recent study showed that this could be due to the upregulated expression of the IL2 receptor and the downregulation of the transcription factor HELIOS, leading to increased STAT5 phosphorylation (Zhang et al., 2023). Immunosenescent T cells not only express high levels of cytotoxic molecules and inflammatory mediators like IFN‐γ and TNF‐α indicative of a SASP. CD4^+^CD28null of RA patients also significantly upregulate the expression of typical SASP molecules such as the metalloproteinase MMP9 and growth factors like VEGFA, IGF1, and hepatocyte growth factor in response to IL‐15 stimulation, in contrast to their CD28^+^ counterparts (Igarashi et al., 2022; Rioseras et al., 2021). In addition, CD28null cells *upregulate receptors that are typical for the innate immune system*: killer immunoglobulin‐like receptors (KIR) such as *CD57* (Leu7) and killer‐cell lectin‐like receptor G1 (*KLRG1*) (Pereira & Akbar, 2016). The acquisition of these receptors, typically expressed by NK cells, allows immunosenescent T cells to exert antigen‐independent effector functions. In conclusion, immunosenescent T cells acquire innate‐like immune functions and display a distinct secretory phenotype that distinguishes them from CD28^+^ T cells.

#### 3.2.5 Loss of functions in T cells

Another hallmark of senescence is the (partial) loss of cell‐specific functions. CD28null T cells obviously lose CD28‐mediated functions, such as co‐stimulation upon T cell receptor engagement and IL‐2 production. Nevertheless, as described in the previous paragraph, they retain multiple effector functions. In contrast, another subpopulation of memory T cells, the above‐mentioned *exhausted T cells*, loses its memory effector functions, such as a high proliferative capacity or the ability to respond to antigenic challenge with cytokine production or cytotoxicity (Lee et al., 2016; Wherry, 2011). Both immunosenescent and exhausted T cells are memory T cells that have undergone persistent antigen stimulation. But while immunosenescent T cells become resistant to apoptosis, exhausted T cells are programmed to undergo apoptosis as they express high levels of programmed cell death protein‐1 (*PD‐1*) (Janelle et al., 2021). Single‐cell RNA seq of T cell receptors showed that clonal expansion is responsible for the age‐associated increase in PD1^+^CD8^+^ T cells in mice (Mogilenko et al., 2021). PD‐1 expression is mainly upregulated on early differentiated T cells: naïve and central memory T cells, but is down‐regulated during late stages of differentiation (Sauce et al., 2007). Markers of cellular senescence have not been investigated in exhausted T cells to the same extent as in immunosenescent T cells. The main differences between immunosenescent and exhausted T cells with regard to the hallmarks of senescence are summarized in Table 1 and depicted in Figure 1. Although literature often describes immunosenescent and exhausted T cells as subsets with distinct markers and properties, it should be noted that they are not mutually exclusive, as markers of immunosenescence and exhaustion can be co‐expressed (Song et al., 2018).

Exhausted T cells are detected after chronic viral infections (Moskophidis et al., 1993). As they lose effector functions and become highly susceptible to apoptosis, this leads to a reduction in the numbers and efficacy of those cells that should attack the virus‐infected cells, allowing the persistence of the virus. Exhausted T cells can further be distinguished by a high expression of inhibitory receptors such as T cell immunoglobulin mucin‐3 (*TIM‐3*), cytotoxic T lymphocyte‐associated protein 4 (*CTLA‐4*), and T‐cell immunoglobulin and immunoreceptor tyrosine‐based inhibitory motif (ITIM) domain (*TIGIT*) (Lee et al., 2016; Song et al., 2018). Expression of these inhibitory receptors leads to the suppression of T cell effector functions (Sakuishi et al., 2010).

T cell exhaustion is a phenomenon that receives particular attention in the field of cancer immunotherapies. For hematological cancers, *chimeric antigen receptor* (*CAR*) *T* cells can be produced that express a genetically engineered T cell receptor specifically targeting the tumor antigens. However, for solid tumors, CAR T cell therapy has been less successful. This is partly due to the inability of T cells to infiltrate the tumor but also due to T cell exhaustion caused by the strong immunosuppressive features of the tumor microenvironment (Johnson et al., 2022; van der Heide et al., 2022). Interestingly, the dysfunction of exhausted T cells can be reversed by immune checkpoint inhibitors, such as anti‐PD1 and anti‐CTLA4, which holds promise to boost immune responses against cancer cells. Some senescent cells express the PD‐1 Ligands PD‐1 L or PD‐2 L. PD‐1 L expressing senescent cells accumulate with aging (Wang et al., 2022). PD‐1 Ligands prevent cytotoxicity induced by PD‐1‐expressing T cells. As such, PD‐1 L and PD‐2 L‐expressing senescent cells are resistant to T cell‐mediated immune surveillance, even though they secrete high levels of SASP. Consequently, anti‐PD‐1 treatment results in increased T‐cell mediated elimination of PD‐1 L^+^ senescent cells in aging mice and in a preclinical model of steatohepatitis (Onorati et al., 2022; Wang et al., 2022). Likewise, targeting PD‐L2 combined with chemotherapy leads to improved CD8 T cell‐mediated eradication of senescent cancer cells (Chaib et al., 2024).

It seems that PD1^+^ T cells can become senescent as well. After repeated proliferation in culture, PD‐1 expressing human T cells show DNA damage, cease proliferation and upregulate expression of p16 (Janelle et al., 2021).

#### 3.2.6 Altered gene expression, impaired mitochondria and increased SA‐β‐gal activity in T cells

Senescent cells typically display epigenetic changes, such as chromatin remodeling, altered methylation patterns and histone modifications, thereby altering gene expression patterns and changing the metabolic functions in senescent cells (Crouch et al., 2022). An example of epigenetic changes in senescent cells are the SAHFs, that have a repressive H3K9‐Me3 core segregated in space from an H3K27me3 ring, forming high‐order chromatin structures (Chandra & Narita, 2013). A few studies analysed chromatin accessibility in T cells isolated from young and older individuals. They found a loss of accessibility in gene promoter regions of the nuclear respiratory factor 1 (NRF1) in naïve CD8^+^ T cells of aged individuals (Moskowitz et al., 2017) NRF1 is a transcription factor that regulates gene expression of mitochondrial respiratory chain proteins. In addition, memory CD8^+^ T cells of aged individuals display a decreased chromatin accessibility in promoters and enhancers associated with IL‐7R signaling (Ucar et al., 2017). Finally, naïve CD4^+^ T cells from aged individuals show altered chromatin accessibility resulting in reduced HELIOS expression and aberrant IL‐2 receptor signaling, associated with increased inflammatory effector functions (Zhang et al., 2023). Although these studies did not directly measure senescence‐associated heterochromatin foci (SAHF) or H3K9‐Me3 in immunosenescent or exhausted T cells, they support a link between chromatin remodeling and T cell aging.

Another hallmark of senescence is the accumulation of dysfunctional mitochondria. For T cells, there appears to be a difference between immunosenescent CD8^+^ versus CD4^+^ T cells. CD8^+^ TEMRA cells have impaired mitochondrial function with elevated ROS production, similar to what has been described for senescent fibroblasts (Henson et al., 2014). On the other hand, CD4^+^ TEMRA cells have healthier mitochondria (Callender et al., 2020). Exhausted T cells also have impaired mitochondrial function. Recent studies indicate that the combination of chronic antigen stimulation with severe metabolic stress, such as hypoxia, can lead to mitochondrial dysfunction in T cells (Scharping et al., 2021). This, in turn, leads to the production of intolerable levels of ROS, which subsequently promotes T cell exhaustion (Scharping et al., 2021). It is noteworthy that T cells with dysfunctional mitochondria, due to deficiency in the mitochondrial transcription factor A (TFAM), can induce senescence in other organ systems. They also cause inflammaging and premature death (Desdin‐Mico et al., 2020). These studies further suggest that dysfunctional T cells may drive senescence and age‐related pathologies throughout the body. In conclusion, highly differentiated CD8^+^ T cells and exhausted T cells display impaired mitochondrial function.

*Senescence‐associated β‐Gal activity* significantly increases in CD4^+^ and to a higher degree in CD8^+^ T cells in older individuals (Martinez‐Zamudio et al., 2021). The CD8^+^ T cells marked by high β‐Gal activity also show other markers of senescence, namely decreased proliferation potential, an upregulation of p16 and p21, as well as double‐stranded DNA breaks, also in the telomere regions. Thus, T cells with the hallmarks of cellular senescence significantly increase in elderly individuals cellular senescence mostly arises in highly differentiated T cells, but to a lesser extent, also in central memory T cells and even in naïve T cells (Martinez‐Zamudio et al., 2021). In line with this, telomere attrition and telomere‐associated DNA damage have previously been described in naïve T cells of RA patients, indicating that even naïve T cells may be vulnerable to cellular senescence (Li et al., 2018). Most exhausted PD1^+^ T cells show low β‐Gal activity, indicating that T cell exhaustion is not indicative of cellular senescence (Martinez‐Zamudio et al., 2021) theHighly differentiated TEMRA cells seem to be most prone to cellular senescence, but senescent T cells may arise in other differentiation stages as well. As such, to identify senescent T cells, one cannot rely on flow cytometry markers like CD57, KLRG1 or CCR7^−^/CD45RA^+^, but should use the classical hallmarks of cellular senescence, such as SA‐β‐Gal and p16.

## 4 POTENTIAL THERAPEUTIC CONSEQUENCES

To address the age‐related accumulation of senescent cells, drugs known as “senolytics” have been developed that selectively eliminate senescent cells. As the immune system is responsible for clearing senescent cells, boosting these immune functions could represent an alternative way to clear senescent cells. Recently, CAR T cells with chimeric antigen receptors specifically engineered to target senescent cells have shown therapeutic potential in mouse models of liver fibrosis and lung adenocarcinoma (Amor et al., 2020). It will be interesting to see if this approach is effective in the treatment of other age‐related pathologies. In addition, preventing or reversing immunosenescence could be another strategy to rejuvenate tissues throughout the body. At present, it is uncertain whether senolytics are able to eliminate senescent T cells. It will be important to assess whether immunosenescent T cells depend on Bcl‐2 family members for their survival. If so, they could be eliminated by senolytics such as Navitoclax or Venetoclax. Another mechanism worth exploring is whether p53 is also a key regulator of immunosenescent T cell viability. If so, the FOXO4‐DRI peptide could be used to break the interaction between FOXO4 and p53 and consequently induce apoptosis in dysfunctional T cells, as was previously shown in senescent fibroblasts (Baar et al., 2017). However, given the limited understanding of the role of senescence in earlier T cell differentiation stages, it may be more desirable to restore the function of the dysfunctional T cells. To this end, various approaches are being investigated. Blocking of KLRG1 signaling boosts the proliferative capacity of CD28null cells (Henson et al., 2009). Inhibition of p38 MAPK signaling boosts the proliferation, telomerase activity, and mitochondrial biogenesis in TEMRA cells (Henson et al., 2015; Lanna et al., 2014). Likewise, blockade of the PD‐1 pathway and shRNA‐mediated p16 targeting can both restore T cell functions in exhausted T cells (Janelle et al., 2021; Lee et al., 2015). Recently, single‐cell RNA sequencing studies have revealed shifts in more low‐abundant immune cell subsets, such as increases in Granzyme K expressing CD8^+^ TEM cells and in type 2 memory T cells with aging (Mogilenko et al., 2021; Terekhova et al., 2023). Ultimately, it will be essential to fully understand the functional changes in the different T cell subpopulations in a specific age‐related disease to design adequate therapies that target the relevant cells.

## 5 CONCLUSION

In young and healthy individuals, damaged cells can enter a state of cellular senescence, which limits the spread of dysfunctional cells. These senescent cells produce a SASP which attracts immune cells that may ultimately clear these senescent cells. Recent studies suggest that when the immune cells themselves become senescent, they fail to clear other senescent cells and drive senescence, and age‐related dysfunction of other organs (Desdin‐Mico et al., 2020; Yousefzadeh et al., 2021).

As we age, T cells may develop cellular senescence, similar to fibroblasts and other cell types in which the state of cellular senescence has been extensively investigated. But importantly, the cell surface markers frequently used to detect immunosenescent T cells, such as the loss of CD27 and CD28 expression or the upregulated KLRG‐1 or Leu7 expression, or the exhaustion marker PD‐1, do not accurately demarcate the population that is in a true state of senescence. Rather, they mark a heterogenous population of T cells, mostly but not exclusively consisting of senescent cells. Referring to this population as senescent T cells is, at best, an oversimplification and leads to a significant underestimation and misinterpretation of the actual number of senescent T cells in individual patients. Several hallmarks of cellular senescence, such as p16, p21, absence of proliferation, DNA‐SCARS, TAFs, SAHFs, loss of LMNB1 and increased senescence‐associated β‐gal activity, should be included to accurately measure senescent T cells.

Future studies should investigate the functional properties of T cells that are in a state of cellular senescence in the different T cell differentiation stages. For a long time, research into T cell senescence has focused on late‐stage differentiation stages, such as the CD28null population, the CD57^+^ TEMRA cells (the effector memory cells that re‐express CD45RA), or the exhausted T cells. The question now arises whether earlier T cell differentiation stages can also become senescent. Knowledge on the functional consequences of accumulating senescent naïve or central memory T cells is lacking. It is essential to determine whether these subpopulations of senescent T cells increase in age‐related diseases and actively contribute to pathology. Additionally, it is important to explore whether they secrete a SASP and how they differ functionally from non‐senescent T cells of the same differentiation stage. These are critical open questions that require further investigation.

In conclusion, the distinct hallmarks of senescence should be applied together with specific markers for the different T cell differentiation stages (CCR7, CD45RA/RO, CD27, and CD28) and the typical markers of immunosenescence (KLRG1 and CD57) and exhaustion (PD‐1, TIM‐3, CTLA‐4, and TIGIT). Further research is needed to fully understand the role of T cell senescence in aging and pathology to design therapies that selectively target those T cells necessary for preventing the accumulation of senescent cells or the development of age‐related pathology.

## AUTHOR CONTRIBUTIONS

H.S.: Manuscript concept design; writing and creation of Figures and Tables, N.V.: Revising of manuscript and creation of Table. P.dK.: Manuscript writing and revising. S.H. and N.H.: Manuscript concept design; revising and funding. All authors have read and agreed to the published version of the manuscript.

## CONFLICT OF INTEREST STATEMENT

P.dK. is Founder, Managing Director and Shareholder of Cleara Biotech B.V.

## Data Availability

Data sharing is not applicable to this article as no new data were created or analyzed in this study.
